# Deoxyelephantopin induces apoptosis via oxidative stress and enhances gemcitabine sensitivity *in vitro* and *in vivo* through targeting the NF-κB signaling pathway in pancreatic cancer

**DOI:** 10.18632/aging.103327

**Published:** 2020-06-11

**Authors:** Daolin Ji, Xiangyu Zhong, Peng Huang, Pengcheng Kang, Kaiming Leng, Wangyang Zheng, Zhidong Wang, Yi Xu, Yunfu Cui

**Affiliations:** 1 Department of Hepatopancreatobiliary Surgery, The Second Affiliated Hospital, Harbin Medical University, Harbin, China; 2 The Key Laboratory of Myocardial Ischemia, Harbin Medical University, Ministry of Education, Harbin, China; 3 Department of Hepatobiliary Surgery, Qingdao Municipal Hospital, Qingdao, China

**Keywords:** deoxyelephantopin, gemcitabine, pancreatic cancer, oxidative stress, NF-κB

## Abstract

Pancreatic cancer is a highly invasive malignant tumor of the digestive system with an unfavorable prognosis worldwide. This trait is thought to be largely attributed to chemoresistance. Chemotherapy is the only hope for patients with advanced pancreatic cancer. Therefore, seeking new effective chemotherapy drugs has become an urgent need. The purpose of our study was to explore whether deoxyelephantopin (DET), a sesquiterpene lactone, has a potential antitumor effect in pancreatic cancer. Additionally, the antitumor effects of DET alone or in combination with gemcitabine (GEM) and the potential mechanism of this combination were revealed. *In vitro* experiments showed that DET suppressed the proliferation, invasion and metastasis of pancreatic cancer cells, induced cell apoptosis via oxidative stress, and enhanced GEM sensitivity by inhibiting the NF-κB signaling pathway. Beyond that, *in vivo* experiments showed that DET not only inhibited pancreatic tumor growth and metastasis but also amplified the antitumor capacity of GEM, which was related to the downregulation of NF-κB and its downstream gene products. In summary, it is possible that DET could be developed as a single agent or combined with conventional chemotherapy drugs to improve the treatment of pancreatic cancer.

## INTRODUCTION

Pancreatic cancer is considered one of the most aggressive and fatal malignant gastrointestinal tract tumors, and its morbidity and mortality rates are gradually increasing [1]. Even with improved diagnostic strategies and the best therapeutic intervention available today, the 5-year survival rate for the patients remains alarmingly dismal, at less than 5% [2, 3]. The latest epidemiological investigation showed that pancreatic cancer accounts for 3% of cancer-related deaths worldwide, making it the 7^th^ most common cause of death induced by cancer worldwide [4]. Surveys in America and Europe showed that pancreatic cancer accounts for 7% of cancer-related deaths and is expected to overtake breast cancer as the 3^rd^ main cause of cancer-related mortality [4, 5]. In China, the incidence of pancreatic cancer is 0.9‰, while the mortality rate is as high as 0.75‰ [6–8]. Due to the unsatisfactory prognosis, the mortality rate is almost equal to the morbidity rate [6–8]. The poor prognosis of pancreatic cancer is extensively linked to its insidious onset, inconspicuous clinical manifestations in the early stage, and lack of effective approaches for early detection and treatment [8]. By the time of definite diagnosis, more than 80% of pancreatic cancer patients are in a locally advanced stage or have distant metastasis, which costs them the opportunity for surgery [8]. Moreover, the five-year survival rate for pancreatic cancer patients undergoing radical resection is only 15-20%, which is still unsatisfactory [8]. GEM is a recognized first-line chemotherapy drug for locally advanced or metastatic pancreatic cancer. However, GEM shows different degrees of adverse reactions and drug resistance during treatment. Hence, the exploration of chemotherapy for pancreatic cancer is ongoing. Seeking novel, less-toxic drugs that can overcome resistance and sensitize cells to traditional chemotherapy agents is still urgently needed.

Gemcitabine (GEM), a nucleoside analogue of deoxycytidine, has been extensively used in a variety of solid tumors since it was approved by the FDA in 1996 [9, 10]. Studies have shown that GEM acts through multiple antitumor mechanisms, including inhibiting DNA synthesis, killing cells in S phase, suppressing the activation of enzymes related to deoxynucleotide metabolism and inducing apoptosis through the caspase pathway [11, 12]. GEM is considered the first- line chemotherapy agent for many malignancies, such as pancreatic cancer, breast cancer and lung cancer [13–16]. Moreover, GEM is also used off-label for multiple types of tumors, and this type of use is sometimes called exploratory treatment [17, 18]. Given the current status of treatment strategies, GEM remains a milestone in adjuvant chemotherapy, neoadjuvant chemotherapy, and palliative therapy for pancreatic cancer patients [19, 20]. Similar to the outcome of treatment with other chemotherapeutic drugs, GEM chemoresistance can occur after the initial therapy is used for a period of time. Although GEM resistance is associated with various genetic and epigenetic changes, the specific mechanisms are still not fully understood and warrant further study. Numerous studies have demonstrated that GEM resistance is closely related to multiple molecular signaling pathways, among which NF-κB, a classical pathway, is particularly important and widely explored [21–23].

NF-κB is composed of a heterodimer of various members of the Rel family that can be classified as NF-κB/Rel proteins, including RelA (p65), RelB, c-Rel, p50 and p52 [24]. The common NF-κB protein refers to the NF-κB1 dimer protein formed by p65/p50 and maintains an inactive state by binding to the inhibitor proteins IκB-α or IκB-β, which inhibit the nuclear transfer of NF-κB [24, 25]. Studies have confirmed that, as a widely expressed intracellular transcription factor, NF-κB is involved in multiple vital cellular functions, such as cell cycle control, apoptosis regulation, stress protection and immune reactions, and it is always abnormally activated in many malignancies [26]. Furthermore, multiple studies have confirmed that NF-κB is in a continuous abnormal activation state in pancreatic cancer cell lines, animal models of cancer and even human tumor specimens [27]. NF-κB activation plays a regulatory role in gene expression, leading to malignant biological phenotypes and chemoresistance [28–30]. In contrast, suppressing abnormal activation of NF-κB significantly enhances the antitumor effect of GEM in pancreatic cancer [29, 30]. In summary, to make progress in pancreatic cancer treatment, the exploration of novel agents that could effectively block the activation of NF-κB is urgently needed.

Natural phytochemicals, promising antitumor agents, have attracted researchers’ attention. Sesquiterpene lactones, natural bioactive compounds, have been shown to possess antitumor and anti-inflammatory potential [31, 32]. *Elephantopus scaber*, a Chinese herbal medicine, is widely applied to treat a variety of diseases, such as diabetes, hepatitis, rheumatism, and infection [32, 33]. Deoxyelephantopin (DET), a natural bioactive sesquiterpene lactone, is extracted from *E. scaber*. In recent years, a growing body of research has elucidated the cytotoxicity of DET and its value in the treatment of malignancies, such as osteosarcoma, cervical cancer, hepatocellular carcinoma, colon carcinoma and breast cancer [32–36]. Studies have indicated that DET not only causes intracellular oxidative stress but also activates the caspase cascade in tumor cells, thereby inducing cell apoptosis through the mitochondrial apoptosis pathway. Moreover, DET has been verified to suppress the abnormal activation of the NF-κB signaling pathway, which plays a critical role in the occurrence and treatment of pancreatic cancer by promoting proliferation, metastasis and GEM chemoresistance and inhibiting apoptosis. Therefore, DET is expected to be used as a novel adjuvant drug in the field of pancreatic cancer chemotherapy. Furthermore, the antitumor effect and potential molecular mechanism of DET in pancreatic cancer need to be further investigated.

In this study, different human pancreatic cancer cell lines and various animal models were used to uncover the functional roles and potential mechanisms of DET *in vitro* and *in* *vivo*. Our results illustrated that DET can inhibit tumor growth and metastasis, induce apoptosis and enhance GEM sensitivity. These effects of DET might be associated with intracellular ROS production, mitochondrial dysfunction, caspase activation and NF-κB signaling pathway inhibition.

## RESULTS

### DET inhibits the proliferation and colony formation of BxPC-3, CFPAC-1 and PANC-1 cells *in vitro*

**Figure 1:**
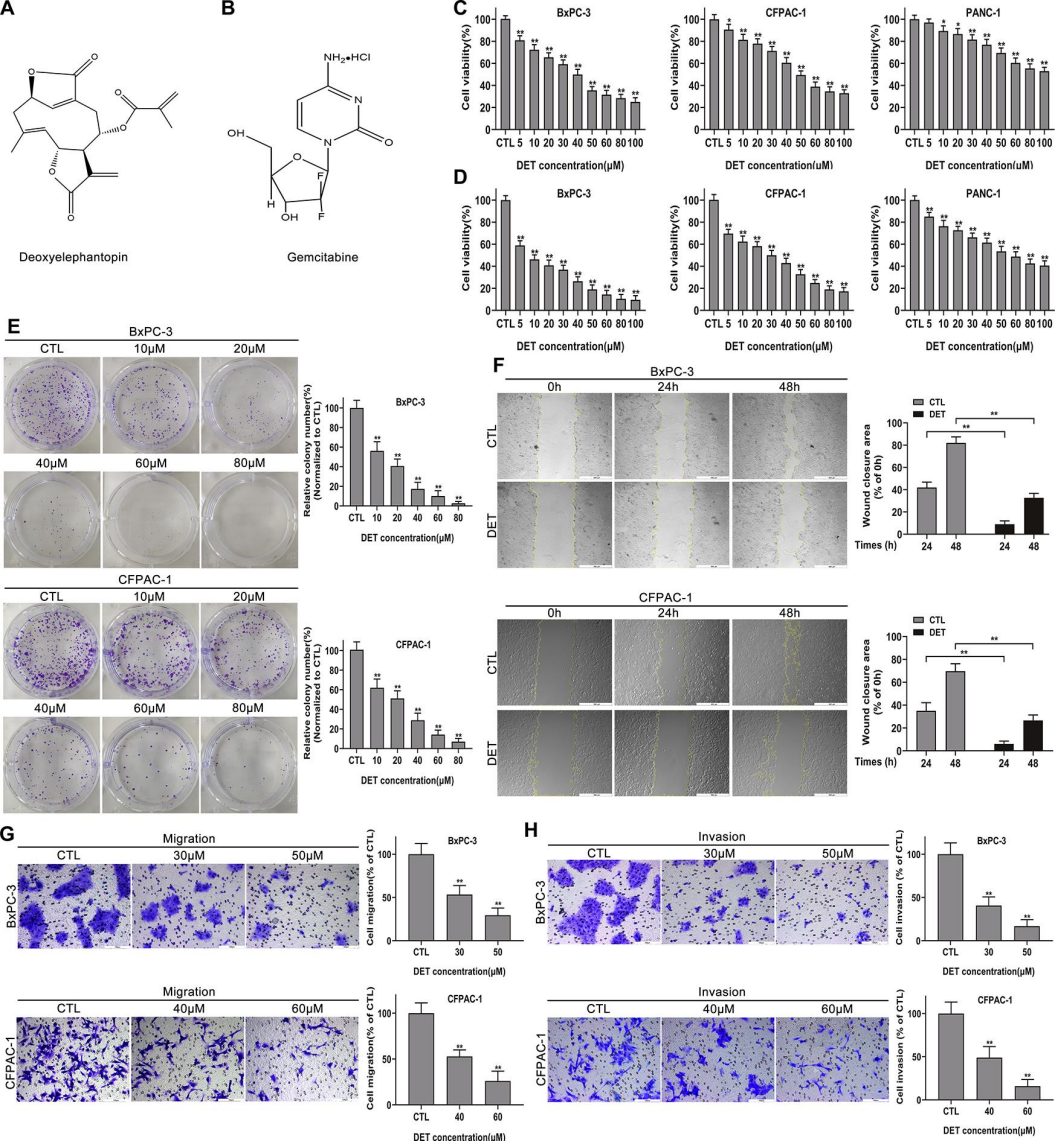
**DET suppressed the proliferation, migration and invasion of pancreatic cancer cells *in vitro*.** (**A**, **B**) The pharmaceutical chemical structures of DET and GEM. (**C**, **D**) Cell viability curves of BxPC-3, CFPAC-1 and PANC-1 cancer cells after DET treatment for 24 h and 48 h were determined by cell counting kit-8 (CCK-8) assays. *P < 0.05, **P < 0.01 versus CTL. CTL, control. (**E**) Colony formation capacities of BxPC-3 and CFPAC-1 cells after DET treatment were evaluated by colony formation assays. **P < 0.01 versus CTL. CTL, control. (**F**) Effect of DET on the migration abilities of BxPC-3 and CFPAC-1 cells were detected by wound healing assays. **P < 0.01 versus CTL. CTL, control. (**G**, **H**) Effect of DET on the migration and invasive abilities of BxPC-3 and CFPAC-1 cells were measured using Transwell assay. **P < 0.01 versus CTL. CTL, control. Magnification, × 40 (**F**), × 200 (**G**, **H**). Scale bar, 500 μm (**F**), 100 μm (**G**, **H**). DET, deoxyelephantopin. GEM, gemcitabine.

After exposure to DET at different concentration gradients ranging from 0 μM to 100 μM for 24 and 48 h, the effect of DET on the proliferation of pancreatic cancer cells was determined by the CCK-8 assay. As shown in Figure 1C, 1D, DET inhibited cell viability, and this effect was not only concentration-dependent but also time-dependent. The determined half maximal inhibitory concentration (IC_50_) value at 24 h was 40 μM for BxPC-3 and 50 μM for CFPAC-1. Moreover, inhibition of cell proliferation was further confirmed through a colony formation assay. The results confirmed that colony formation ability was suppressed by DET in a dose-dependent manner as a result of irreversible damage to cells (Figure 1E).

### DET inhibits the migration and invasion of BxPC-3 and CFPAC-1 cells *in vitro*

The wound healing, Transwell migration and invasion assays were carried out to determine the impact of DET on cell motility. As shown in Figure 1F, pretreatment with DET significantly inhibited the wound closure rate of BxPC-3 and CFPAC-1 cells. In addition, data also showed that DET could markedly suppress the cell migration and invasion capabilities analyzed by the Transwell assay (Figure 1G, 1H).

### DET induces morphological changes in BxPC-3 and CFPAC-1 cells *in vitro*

**Figure 2:**
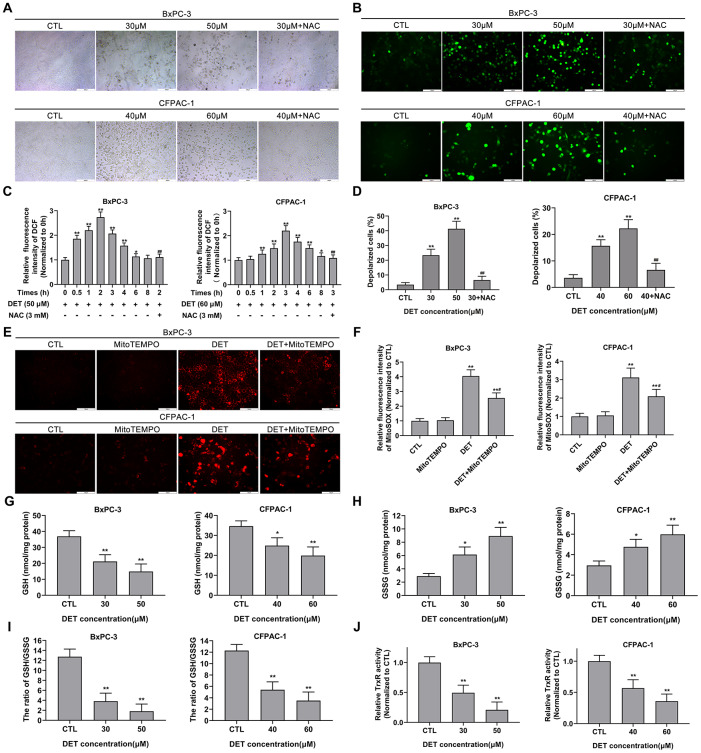
**DET induced oxidative stress, disturbed MMP and unbalanced the REDOX system in pancreatic cancer cells.** (**A**) The DET-induced morphological changes in BxPC-3 and CFPAC-1 cells were observed using microscope. (**B**) Effect of DET on oxidative stress in BxPC-3 and CFPAC-1 cells were evaluated using DCFH-DA probe and observed under fluorescent microscope. (**C**) Effect of DET on fluorescence intensity of DCF were measured using fluorescence microplate reader. *P < 0.05, **P < 0.01 versus CTL. ##*P* < 0.01 versus DET (50 μM) at 2 h or DET (60 μM) at 3 h. CTL, control. (**D**) Effect of DET on MMP in BxPC-3 and CFPAC-1 cells were tested using JC-1 probe and evaluated using fluorescence microplate reader. **P < 0.01 versus CTL. ##*P* < 0.01 versus DET (30 μM) or DET (40 μM). CTL, control. (**E**) Effect of DET on oxidative stress were further assessed using MitoSOX and observed under fluorescent microscope. (**F**) Effect of DET on fluorescence intensity of MitoSOX were detected using fluorescence microplate reader. **P < 0.01 versus CTL. #*P* < 0.05 versus DET single treatment groups. CTL, control. (**G**–**I**) Effect of DET on intracellular GSH, GSSG and the ratio of GSH to GSSG in BxPC-3 and CFPAC-1 cells were assessed using GSSG/GSH quantification kit. *P < 0.05, **P < 0.01 versus CTL. CTL, control. (**J**) Effect of DET on intracellular TrxR activity was measured using thioredoxin reductase assay kit. **P < 0.01 versus CTL. CTL, control. Magnification, ×100 (**A**), × 200 (**B**, **E**). Scale bar, 200 μm (**A**), 100 μm (**B**, **E**). DCFH-DA, 2’, 7’-dichlorofluorescein-diacetate. GSH, reduced glutathione. GSSG, oxidative form of glutathione. TrxR, thioredoxin reductase.

After treatment with DET (30 μM and 50 μM for BxPC-3, 40 μM and 60 μM for CFPAC-1) with or without NAC, the cellular morphological changes were observed using a microscope at ×100 magnification. As shown in Figure 2A, DET induced severe morphological changes in BxPC-3 and CFPAC-1 cells, including a reduction in cell adhesion ability and loss of normal cell morphology. Nevertheless, the cytotoxicity of DET was eliminated by preconditioning cells with a ROS scavenger (NAC 3 mM) for 2 h.

### DET induces oxidative stress and interferes with MMP in BxPC-3 and CFPAC-1 cells *in vitro*

Oxidative stress, mainly associated with the upregulation of intracellular ROS, is an important factor in apoptosis. Since NAC can protect cells from the toxicity of DET and DET has been reported to induce oxidative stress by upregulating intracellular ROS in human osteosarcoma [35], hepatocellular carcinoma [37], and colorectal carcinoma cell lines [34], the level of oxidative stress after DET treatment was initially evaluated by using a fluorescence microscope. With increasing concentrations of DET, the bright green fluorescence (DCF) was significantly enhanced (Figure 2B). The data indicated that the fluorescence intensity of DCF was upregulated over time after DET treatment (50 μM for BxPC-3, 60 μM for CFPAC-1). In BxPC-3, DET increased the DCF fluorescence intensity as early as 0.5 h of treatment; the intensity reached its maximum at 2 h and then gradually decreased to the same intensity as the untreated group at 6 h (Figure 2C). In CFPAC-1 cells, DET increased the fluorescence intensity of DCF, which reached its maximum at 3 h and then returned to the level of the untreated group at 8 h (Figure 2C). NAC pretreatment inhibited DET-induced oxidative stress. Similar data were obtained in the CCK-8 assay, which indicated that the cytotoxicity of DET was oxidative stress-dependent (Supplementary Figure 1A).

Studies have shown that ROS generation and oxidative stress are closely associated with MMP changes [37]. Accordingly, the effect of DET on MMP was measured. After stimulation with DET, the proportion of polarized cells significantly increased, and this result could be reversed by NAC pretreatment (Figure 2D). These results suggested that DET could significantly interfere with MMP.

Previous studies have indicated that intracellular ROS mainly originate from mitochondria. Therefore, oxidative stress was further assessed by mitochondrion-specific probes. As shown in Figure 2E, 2F, the fluorescence intensity of MitoSOX significantly increased after exposure to DET (30 μM for BxPC-3, 40 μM for CFPAC-1) but was attenuated by pretreatment with a mitochondrion-targeted antioxidant (MitoTEMPO). These results further confirmed that the cytotoxicity of DET was mainly mediated by oxidative stress.

### DET depletes intracellular GSH and inhibits intracellular TrxR activity in BxPC-3 and CFPAC-1 cells

GSH, an antioxidant and free radical scavenger in cells, plays an important role in preventing cells from oxidative damage. In addition, a previous study showed that DET could deplete intracellular GSH in HepG2 cells [37]. Hence, levels of intracellular reduced GSH and GSSG, as well as the ratio of GSH to GSSG, were detected. As shown in Figure 2G, the level of intracellular GSH was significantly decreased after exposure to DET. Conversely, the level of GSSG was upregulated (Figure 2H). Additionally, the ratio of GSH to GSSG was downregulated after stimulation with DET (Figure 2I).

Intracellular TrxR, a key enzyme in the thioredoxin system, is also linked to regulation of the intracellular redox equilibrium and avoidance of oxidative damage. As shown in Figure 2J, intracellular TrxR activation was significantly downregulated after exposure to DET.

### DET induces apoptosis in BxPC-3 and CFPAC-1 cells

**Figure 3:**
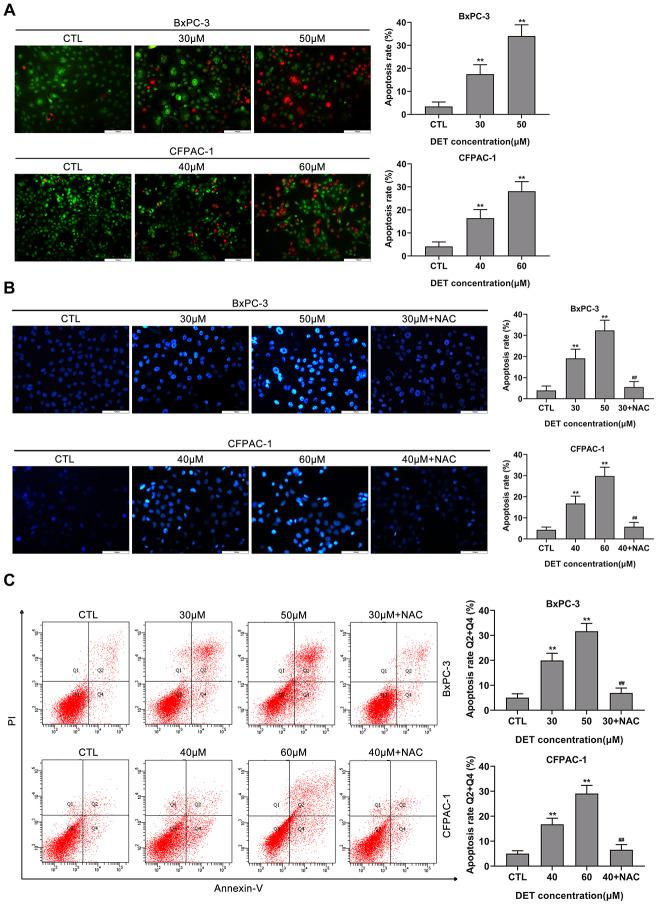
**DET induced apoptosis in BxPC-3 and CFPAC-1 cells *in vitro*.** (**A**) DET-induced apoptosis in BxPC-3 and CFPAC-1 cells were tested using AO/EB double staining assay. **P < 0.01 versus CTL. CTL, control. (**B**) DET-induced apoptosis in BxPC-3 and CFPAC-1 cells were assessed using Hoechst 33342 staining. **P < 0.01 versus CTL. ##*P* < 0.01 versus DET (30 μM) or DET (40 μM). CTL, control. (**C**) DET-induced apoptosis in BxPC-3 and CFPAC-1 cells were determined using Annexin V-FITC/PI double staining. Annexin V-FITC (-) and PI (-) cells were defined as alive, Annexin V-FITC (+) but PI (-) cells were defined as early apoptosis, Annexin V-FITC (+) but PI (+) cells were considered to be late apoptosis. Annexin V-FITC (-) and PI (+) cells were thought to be necrotic cells. The total apoptosis rate was the sum of early apoptosis rate and late apoptosis rate. **P < 0.01 versus CTL. ##*P* < 0.01 versus DET (30 μM) or DET (40 μM). CTL, control. Magnification, × 200 (**A**, **B**). Scale bar, 100 μm (**A**, **B**).

Given the cytotoxicity of DET and its impact on cell morphology, apoptosis was initially verified using AO/EB double-fluorescence staining and Hoechst 33342 staining after exposure to DET. AO/EB staining suggested that apoptotic cells increased significantly after DET treatment, and pretreating cells with NAC reversed apoptosis (Figure 3A and Supplementary Figure 1B). In the Hoechst 33342 staining analysis, DET induced significant nuclear morphological changes in cells, which indicated the upregulation of apoptosis (Figure 3B). Moreover, pretreatment with NAC rescued the original phenotype.

A flow cytometric assay was applied to further evaluate DET-induced apoptosis. The data showed that DET induced apoptosis in BxPC-3 and CFPAC-1 cells with dependence on concentration. In addition, pretreating cells with NAC significantly reversed apoptosis, which implied that DET regulated apoptosis mainly by inducing oxidative stress (Figure 3C).

### DET regulates Bcl-2 family protein expression levels and induces caspase cascade reaction *in vitro*

**Figure 4:**
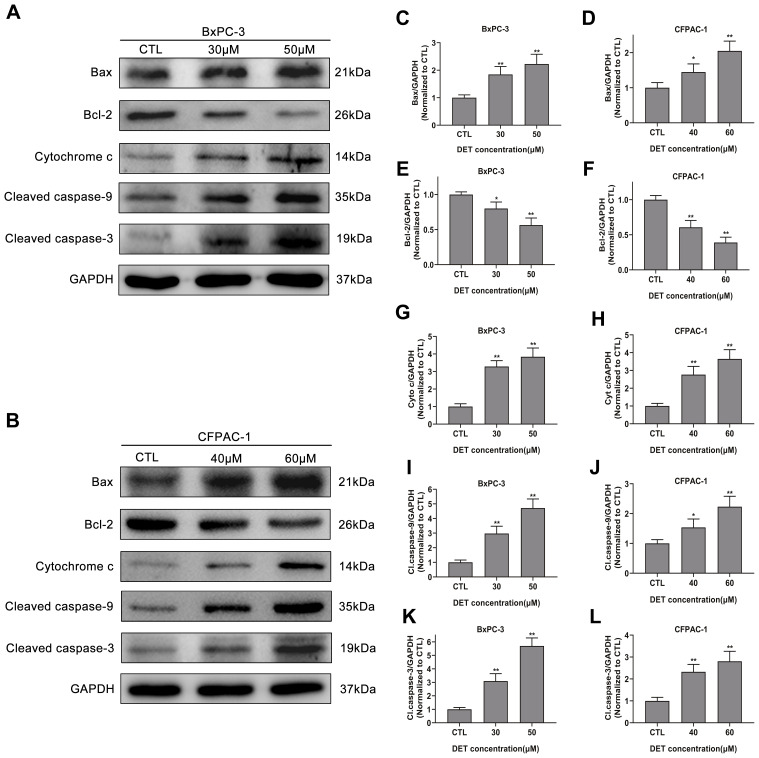
**Effect of DET on apoptosis-related proteins.** (**A**, **B**) DET activated apoptosis-related proteins in BxPC-3 and CFPAC-1 cells in a concentration dependent manner. Quantitative statistics of immunoblotting analysis for Bax levels (**C**, **D**), Bcl-2 levels (**E**, **F**), cytochrome c levels (**G**, **H**), cleaved caspase-9 levels (**I**, **J**), cleaved caspase-3 levels (**K**, **L**), *P < 0.05, **P < 0.01 versus CTL. CTL, control.

Since DET can induce oxidative stress and disturb MMP, the upstream protein expression of the mitochondrial apoptosis pathway was evaluated by immunoblotting (Figure 4A, 4B). Experimental data indicated that DET upregulated Bax expression (Figure 4C, 4D) and downregulated Bcl-2 expression (Figure 4E, 4F), thereby promoting the release of cytochrome c from mitochondria into the cytoplasm (Figure 4G, 4H).

As caspase-9 and caspase-3 are downstream key transporters and executors of the mitochondrial apoptosis pathway and are activated by cleavage, the cleaved forms of these proteins were analyzed. The results demonstrated that DET upregulated cleaved caspase-9 (Figure 4I, 4J) and cleaved caspase-3 expression (Figure 4K, 4L), indicating activation of the caspase cascade. Moreover, pretreatment with 100 μM z-VAD-fmk, a pancaspase inhibitor, almost completely eliminated the toxicity of DET (Supplementary Figure 1B).

### DET suppresses constitutive and gemcitabine-induced NF-κB activation in BxPC-3 cells

**Figure 5:**
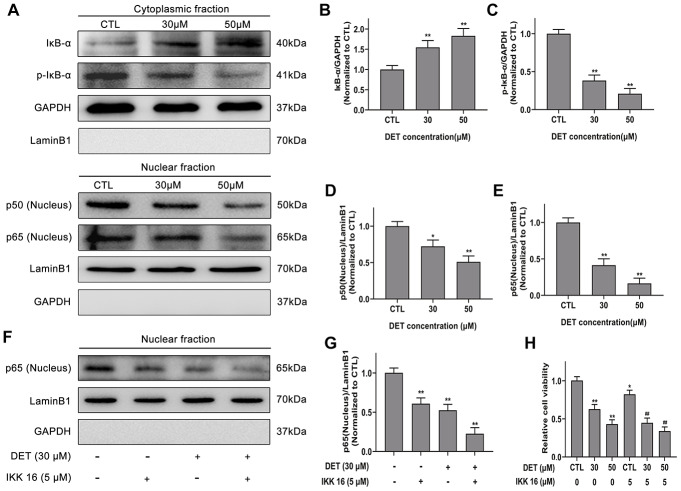
**Effect of DET on NF-κB activity in gemcitabine-resistant BxPC-3 cell line.** (**A**) Effect of DET on NF-κB activity. Quantitative statistics of immunoblotting assays for IκB-α levels (**B**), p-IκB-α levels (**C**), NF-κB-p50 levels (**D**), NF-κB-p65 levels (**E**). *P < 0.05, **P < 0.01 versus CTL. CTL, control. (**F**) Effect of DET and IKK 16 on NF-κB activity. (**G**) Quantitative statistics of immunoblotting assays for NF-κB-p65 levels. **P < 0.01 versus CTL. CTL, control. (**H**) Effect of DET and IKK 16 on cell viability. *P < 0.05, **P < 0.01 versus CTL. ##*P* < 0.01 versus CTL added IKK 16. CTL, control.

A previous study showed that abnormally activated NF-κB signaling is closely linked to chemoresistance. Therefore, we verified whether DET could suppress its activation to reverse chemoresistance. NF-κB expression after DET treatment was examined. Data confirmed that the expression of p50 and p65 was significantly downregulated after DET treatment in a concentration-dependent manner (0 μM, 30 μM, 50 μM), indicating that DET could block the p50/p65 dimer (the main structure of NF-κB) translocating from the cytoplasm to the nucleus (Figure 5A). This block of translocation of NF-κB was further verified to be associated with the inhibition of IκB-α phosphorylation, mainly via upregulated expression of IκB-α and downregulated expression of p-IκB-α (Figure 5A). Protein expression differences of IκB-α, p-IκB-α and NF-κB-p50/p65 are shown in Figure 5B–5E.

IκB kinase inhibitor 16 (IKK 16), a selective IKK inhibitor, can effectively suppress the translocation of NF-κB-p65. As the experimental data show in Figure 5F, 5G, DET elicited an effect similar to that of IKK 16. Thus, the cytotoxicity of IKK 16 combined with DET in BxPC-3 cells was determined by CCK-8 assay. The results indicated that the cytotoxicity of DET was increased significantly after combined treatment with IKK 16 (Figure 5H).

**Figure 6:**
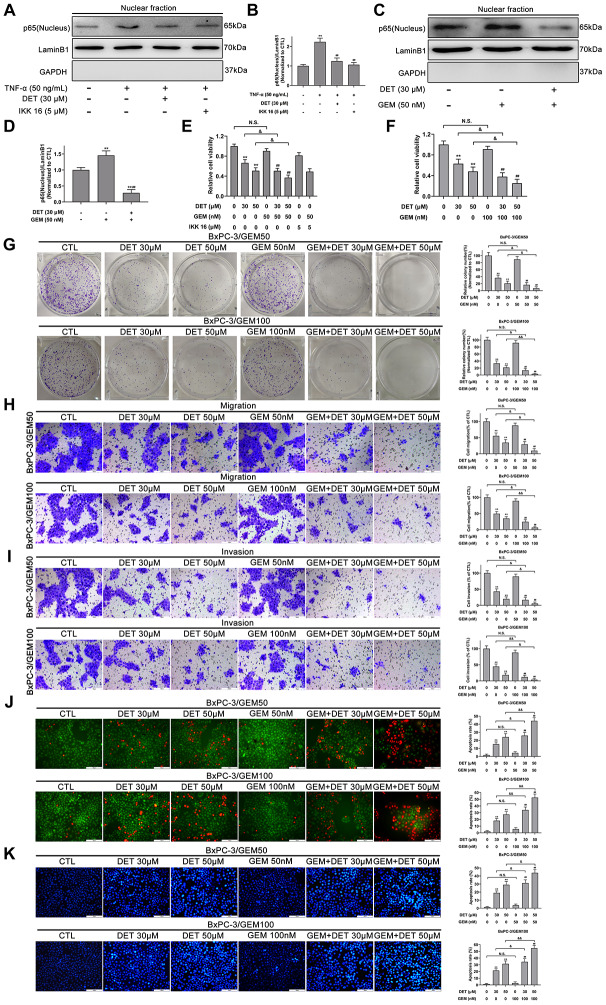
**DET inhibited the TNF-α and GEM induced NF-κB activity and sensitized the antitumor effect of GEM *in vitro*.** (**A**) DET inhibited TNF-α-induced NF-κB activity. (**B**) Quantitative statistics of immunoblotting assays for NF-κB-p65 levels. **P < 0.01 versus CTL. ##*P* < 0.01 versus TNF-α single treatment group. CTL, control. (**C**) DET inhibited GEM-induced NF-κB activity. (**D**) Quantitative statistics of immunoblotting assays for NF-κB-p65 levels. **P < 0.01 versus CTL. ##*P* < 0.01 versus GEM single treatment group. CTL, control. (**E**) Reduction of cell viability after 24 h combined treatment of DET and GEM, and IKK 16 and GEM in BxPC-3/GEM50 cell line. **P < 0.01 versus CTL. ##*P* < 0.01 versus GEM single treatment group. &*P* < 0.05. CTL, control. (**F**) DET sensitized cells to the inhibitory effects of GEM on cell viability in BxPC-3/GEM100 cell line. **P < 0.01 versus CTL. ##*P* < 0.01 versus GEM single treatment group. &*P* < 0.05. CTL, control. (**G**) DET enhanced the inhibitory effect of GEM on cell proliferation in GEM-resistant cell lines. **P < 0.01 versus CTL. ##*P* < 0.01 versus GEM single treatment group. &*P* < 0.05, &&*P* < 0.01. CTL, control. (**H**, **I**) DET reinforced the suppression of GEM on the migration and invasion of GEM-resistant BxPC-3 cell lines. **P < 0.01 versus CTL. ##*P* < 0.01 versus GEM single treatment group. &*P* < 0.05, &&*P* < 0.01. CTL, control. (**J**, **K**) DET amplifies the role of GEM in inducing apoptosis in GEM-resistant BxPC-3 cell lines. **P < 0.01 versus CTL. ##*P* < 0.01 versus GEM single treatment group. &*P* < 0.05, &&*P* < 0.01. CTL, control. Magnification, × 200 (**H**–**K**). Scale bar, 100 μm (**H**–**K**).

TNF-α, an important regulator of inflammation and immunity, can activate the NF-κB signaling pathway. Therefore, NF-κB-p65 expression induced by TNF-α (50 ng) with or without DET (30 μM) was measured using Western blot analysis. The data showed that DET could suppress the translocation of NF-κB-p65, showing the same effect as IKK-16 (Figure 6A, 6B). A large number of investigations have confirmed that GEM activates NF-κB during the treatment of pancreatic cancer, which is closely related to chemoresistance [38]. Our study confirmed that DET inhibited GEM-induced NF-κB activation, mainly by suppressing the nuclear translocation of p65 (Figure 6C, 6D). Since GEM induced the abnormal activation of NF-κB, DET and IKK 16 could inhibit this process, the cytotoxicity of GEM combined with DET was detected. As shown in Figure 6E, DET, similar to IKK 16, sensitized cells to GEM. Taken together, the above data suggest that DET inhibits NF-κB activation induced by either intracellular or extracellular stimuli, and this mechanism is closely associated with DET-mediated cell apoptosis and sensitizes cancer cells to GEM.

### DET improves the antitumor effect of GEM *in vitro*

The reversal effect of DET on chemoresistance of GEM was evaluated in GEM-resistant cell lines (BxPC-3/GEM50 and BxPC-3/GEM100) by CCK-8, colony formation, Transwell, AO/EB and Hoechst 33342 assays. As shown in Figure 6E–6G, DET improved the inhibitory effects of GEM on cell viability and colony formation. Additionally, the suppression of migration and invasion by GEM in GEM-resistant BxPC-3 cells was reinforced by DET (Figure 6H, 6I). In addition, DET improved the role of GEM in inducing apoptosis in GEM-resistant BxPC-3 cell lines. (Figure 6J, 6K).

### DET enhances the inhibitory effect of GEM on tumor growth *in vivo*

**Figure 7:**
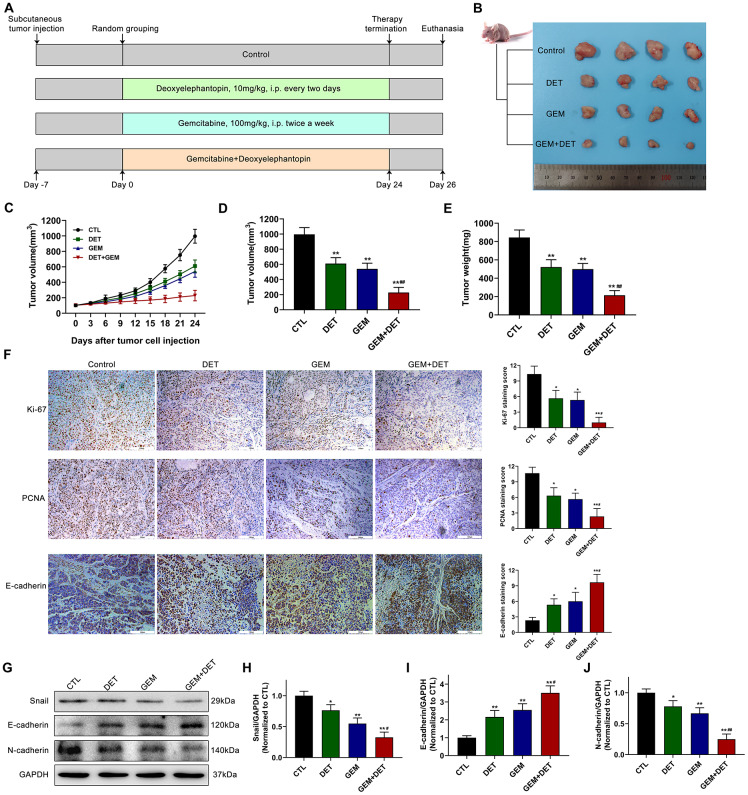
**DET amplified the effect of GEM to inhibit the growth of pancreatic cancer *in vivo*.** (**A**) The specific grouping strategies and treatment patterns of subcutaneous tumor model. (**B**) Xenograft tumors were established by subcutaneous injection of BxPC-3 cells (n = 4). (**C**, **D**) Curves of subcutaneous tumor volume in nude mice. **P < 0.01 versus CTL. ##*P* < 0.01 versus GEM monotherapy group. CTL, control. (**E**) The final quantitative statistics of tumor weight. **P < 0.01 versus CTL. ##*P* < 0.01 versus GEM monotherapy group. CTL, control. (**F**) Ki-67, PCNA and E-cadherin staining and quantitative statistics of the xenograft tumors were shown. *P < 0.05, **P < 0.01 versus CTL. #*P* < 0.05 versus GEM monotherapy group. CTL, control. (**G**) EMT-related protein levels in tumor tissues were detected and quantified by western blotting. Quantitative statistics of western blotting analysis for Snail levels (**H**), E-cadherin levels (**I**) and N-cadherin levels (**J**). *P < 0.05, **P < 0.01 versus CTL. #*P* < 0.05, ## *P* < 0.01 versus GEM monotherapy group. CTL, control. Magnification, × 200 (**F**). Scale bar, 100 μm (**F**).

Based on the above experimental results, the inhibitory effects of DET and GEM alone or together on tumor growth were determined in a subcutaneously implanted nude mouse tumor model. The specific grouping strategies and treatment patterns are listed in Figure 7A. The data indicated that DET increased the inhibitory effect of GEM on tumor growth (Figure 7B–7D). The tumor volume in the GEM combined with DET group is lower than that in the untreated control group at the end of treatment; in addition the tumor growth rate was slower than that in the DET or GEM monotherapy group (Figure 7C). DET and GEM alone had similar reductions in tumor burden (Figure 7C, 7D). Additionally, the final tumor weight in the GEM combined with DET group was lower than that in the other three groups, which was consistent with the results of tumor volume analysis (Figure 7E). Moreover, the IHC assay indicated that the levels of proliferation-related protein (Ki-67 and PCNA) were lower and the level of EMT-related protein (E-cadherin) was higher in tumor specimens of the GEM combined with DET group compared with those of the other three groups (Figure 7F).

Epithelial-mesenchymal transition (EMT) is closely linked with tumor progression and metastasis. Therefore, the expression of EMT-related proteins in tumor tissue was evaluated by immunoblotting (Figure 7G). Research data showed that the expression of Snail (Figure 7H) and N-cadherin (Figure 7J) was downregulated in the DET monotherapy, GEM monotherapy and GEM combined with DET therapy groups compared to the no treatment control group. By contrast, the E-cadherin expression level was upregulated (Figure 7I).

### DET amplifies the GEM effect against liver and lung metastasis of pancreatic cancer, extends survival time in mice with lower side effects

**Figure 8:**
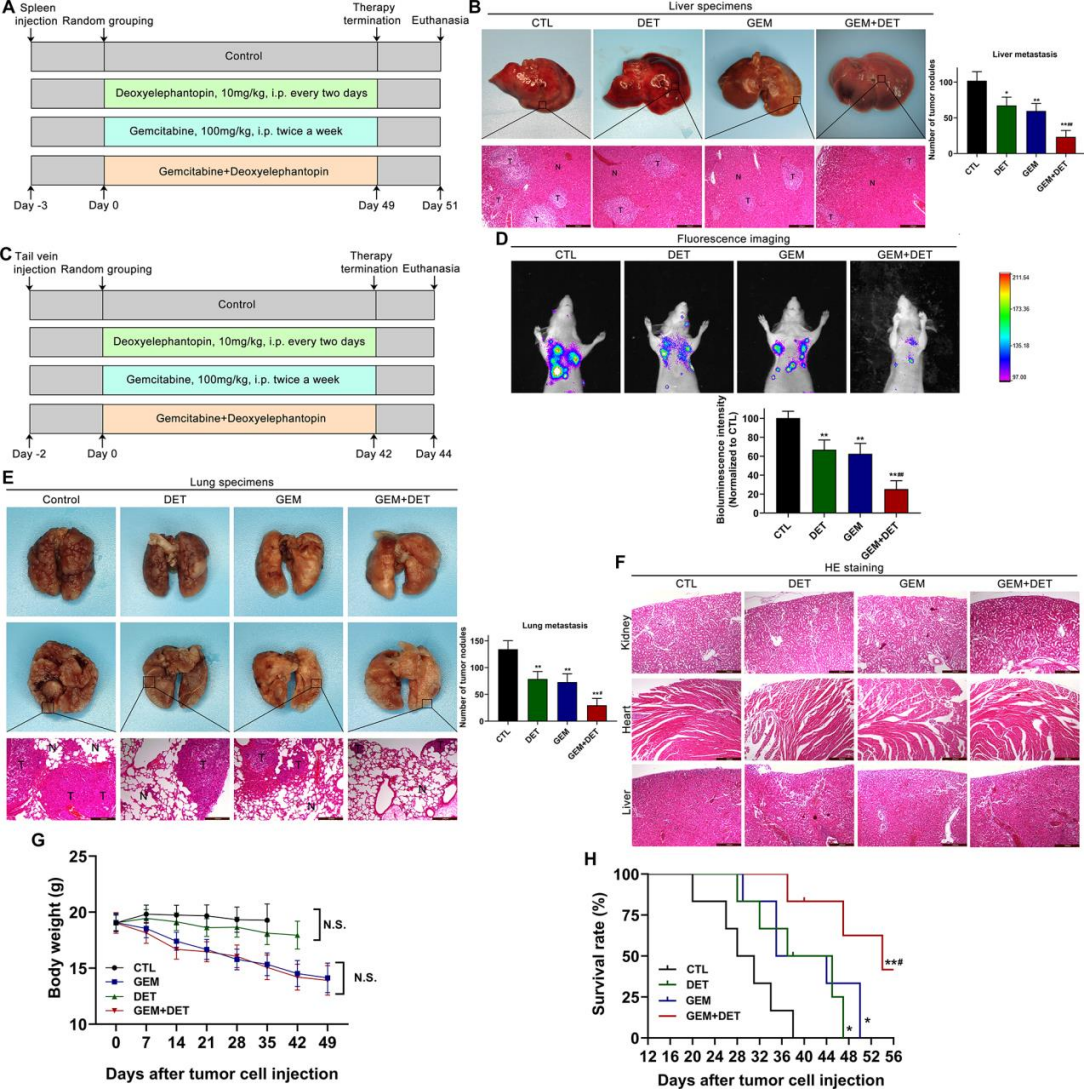
**DET amplified the effect of GEM on suppressing metastasis of pancreatic cancer *in vivo*.** (**A**) The specific grouping strategies and treatment patterns of liver metastasis model. (**B**) Experimental liver metastasis animal model was obtained by injecting BxPC-3 cells in the spleen of nude mice. *P < 0.05, **P < 0.01 versus CTL. ##*P* < 0.01 versus GEM monotherapy group. CTL, control, n = 5. (**C**) The specific grouping strategies and treatment patterns of lung metastasis model. (**D**) Therapeutic effect was measured using an *in vivo* luminescence imaging system. **P < 0.01 versus CTL. ##*P* < 0.01 versus GEM monotherapy group. CTL, control, n = 5. (**E**) Experimental lung metastasis animal model was obtained by injecting luciferase-labeled BxPC-3 cells via the tail vein of nude mice. **P < 0.01 versus CTL. #*P* < 0.05 versus GEM monotherapy group. CTL, control. (**F**) Heart, kidney and liver tissue structure of nude mice in lung metastasis model. (**G**) Average body weights of the tested BALB/c mice in each treatment group. (**H**) Long rank analysis of survival rate of mice in each treatment group. *P < 0.05, **P < 0.01 versus CTL, #*P* < 0.05 versus GEM monotherapy group. CTL, control, n = 5. Magnification, × 100 (**B**, **E**, **F**). Scale bar, 200 μm (**B**, **E**, **F**).

To further evaluate the effects of DET on metastasis, a liver metastasis model and a lung metastasis model were established. The detailed treatment schedule for the liver metastasis model is shown in Figure 8A. Experimental results indicated that DET improved the GEM effect against liver metastasis (Figure 8B). For the lung metastasis model, the treatment schedule is shown in Figure 8C. The curative effects were examined by an *in vivo* luminescence imaging system. The data showed that the bioluminescence intensity of the GEM combined with DET group was obviously lower than that of the other three groups. In addition, the bioluminescence intensity between the GEM monotherapy group and the DET monotherapy group was similar but still lower than that of the control group (Figure 8D). After imaging, the lung tissues were dissected. Experimental results showed that the number of pulmonary tumor metastasis in the GEM combined with DET group was significantly lower than that in the other three groups, which was consistent with the above data (Figure 8E). A histopathological assay was carried out by H&E staining to identify the difference in alveolar structure between each treatment group. The typical alveolar structure was retained in the GEM combined with DET group; conversely, the control group showed numerous and fully grown masses with enlarged and irregular nuclei (Figure 8E). All the data suggested that the inhibitory effect of GEM on lung metastasis was significantly enhanced by DET.

In addition, this study preliminarily explored the safety of DET *in vivo*. The hearts, livers and kidneys dissected from the mice in the lung metastasis model were analyzed by H&E staining. The tissue structures were not different between the DET monotherapy group and the control group, indicating that DET had characteristics of high safety and practicability (Figure 8F).

To evaluate the effects of DET on survival time and quality of life, a lung metastasis model was applied as described above. Comparison of weight changes between each group showed that no significant weight loss occurred in the control group, while all the other three groups showed different degrees of weight loss by the end of experiment. Compared with the GEM monotherapy group, mice in the DET monotherapy group had less weight loss. In addition, mice in the GEM combined with DET group showed the most significant weight loss, but there was no significant difference when compared with the mice in the GEM monotherapy group (Figure 8G). Based on the above results, DET enhanced the sensitivity of GEM to inhibit metastasis without worsening the side effects of treatment, indicating that the drug safety of DET was satisfactory. In addition, the results indicated that the survival time of mice in the GEM combined with DET group was significantly prolonged compared with that in the other groups, which further confirmed the previous experimental results. (Figure 8H).

## DISCUSSION

Pancreatic cancer is a highly lethal malignancy characterized by difficulty in early diagnosis and predisposition to local progression and distant metastasis [4]. Despite use of the best treatment available, the prognosis for pancreatic cancer remains dismal. For the majority of patients diagnosed past the early stage who do not have the opportunity for radical surgery, chemotherapy remains an indispensable method. Pancreatic cancer is characterized by dense stroma and deficient vascularization, leading to inadequate drug delivery and intrinsic chemoresistance [39]. GEM is an accepted chemotherapy agent for pancreatic cancer. However, similar to other chemotherapeutic agents, resistance and adverse reactions occur during chemotherapy, substantially reducing the treatment effect. Chemoresistance to GEM occurs through intrinsic resistance and acquired resistance that develops gradually during treatment cycles; some pathways have been confirmed to be linked with chemoresistance [39]. To date, various signaling pathways, such as the NF-κB, PI3K/Akt, Wnt, and Hedgehog pathways, which are associated with cell proliferation, migration, invasion and apoptosis, have been discovered. Among these findings, the NF-κB pathway, discovered earlier and studied extensively, has been reported to be closely related to GEM chemoresistance and is expected to be a potential therapeutic target for pancreatic cancer [40]. The exploration of novel antitumor agents that could not only inhibit the activation of NF-κB but also sensitize cells to GEM and target the malignant behavior of pancreatic cancer to improve the current treatment effect is urgently needed. In this study, we investigated the antitumor effect of DET on malignant biological behavior and GEM sensitivity in human pancreatic cancer cell lines.

*Elephantopus scaber* L. (compositae) is a common herb used in traditional Chinese medicine for many diseases. In current practice, *E. scaber* is mainly used to treat diabetes, diarrhea and inflammation [32]. With the development of pharmaceutical technology, DET, extracted from *E. scaber*, has been found to possess antitumor activity in several kinds of malignant tumors [32, 34, 35, 37]. However, to date, the effect of DET on pancreatic cancer has not been reported.

Previous studies confirmed that tumor cells exhibit increased oxidative stress compared to normal cells due to the imbalance of the oxidation-reduction system, which is associated with multiple cellular biological processes, such as proliferation, differentiation and drug resistance [41, 42]. Recent investigations have shown that sustained oxidative stress could in turn damage tumor cells, so this biological characteristic is expected to serve as a therapeutic target [42, 43]. Our research verified that DET could induce excessive oxidative stress in pancreatic cancer cells. Prior studies found that oxidative stress is often accompanied by a decline in the activity of the antioxidant stress system. Furthermore, GSH and TrxR are considered to be two important intracellular antioxidant systems that are involved in scavenging free radicals and antioxidation [41]. Our results showed that the ratio of GSH to GSSG and the activity of the Trx system were significantly downregulated after DET treatment, indicating that the cytotoxicity of DET was indeed mediated by oxidative stress in pancreatic cancer.

The mitochondrial electron leak in the respiratory chain is the main source of endogenous ROS [44]. The increase in intracellular ROS levels damages mtDNA and leads to transcription disorders of genes related to electron transport in the respiratory chain and ATP production, which further advances oxidative stress [44]. Therefore, this process leads to the disruption of the mitochondrial respiratory chain and eventually lead to the collapse of MMP. In this experiment, we found that DET disturbed MMP, suggesting that DET was responsible for mitochondrial damage.

Apoptosis is a normal programmed death process controlled by a genetic mechanism, and apoptosis is involved in cell renewal and stabilization of the internal environment [45]. Since tumor occurrence is usually associated with disorders of apoptosis, many antitumor drugs exert cytotoxicity by inducing apoptosis. Studies have confirmed that mitochondrion-mediated apoptosis is the main intracellular apoptosis pathway, in which the production of ROS and the destruction of MMP are two early key steps [46]. Additionally, oxidative stress first regulates the expression of apoptosis-related proteins [46, 47]. Our results showed that DET promoted Bax expression and inhibited Bcl-2 expression, which is in line with the abovementioned findings. Expression changes of the above two proteins resulted in enhanced mitochondrial membrane permeability. Next, cytochrome c and apoptosis inducing factor (AIF) were both released from mitochondria to the cytoplasm. Finally, a complex consisting of apoptotic protease activating factor 1 (APAF-1) and cytochrome c further enlisted procapase-9 to activate the caspase cascade, eventually triggering apoptosis [45]. The present research verified that DET promoted cytochrome c release and increased the level of cleaved caspase-9 and cleaved caspase-3. Cell vitality could be partially rescued by a pancaspase inhibitor (z-VAD-fmk), indicating that DET plays an antitumor role in pancreatic cancer mainly through mitochondrion-mediated apoptosis.

Accumulating evidence has confirmed that the NF-κB pathway is closely correlated with GEM chemoresistance in pancreatic cancer [26, 27]. In normal resting cells, the NF-κB dimer, bound to the IκBs, is located in the cytoplasm and remains inactive [24, 25]. However, the NF-κB signaling pathway is activated in pancreatic cancer, breast cancer and colorectal cancer after GEM treatment [40, 48, 49]. The NF-κB signaling pathway is commonly activated due to the activation of the inhibitor of the kappa B kinase complex (IKK), which normally inhibits IκBs by downregulating phosphorylation [24, 25]. Activated IKK promotes the phosphorylation of IκBs and separates IκBs from NF-κB dimers. Next, the free NF-κB dimers translocate into the nucleus and play a part in the transcriptional regulation of genes associated with anti-apoptosis, chemoresistance, inflammation and metastasis [24, 25]. Moreover, studies have shown that inhibition of GEM-induced activation of NF-CB can amplify the antitumor effect of GEM [48, 49]. Therefore, a resistant cell line was established to validate the effect of DET in combination with GEM. The results showed that GEM promoted the translocation of NF-κB from the cytoplasm to the nucleus; conversely, this procedure could be suppressed by DET. In addition, the inhibitory effect of DET on NF-κB activation, which is induced by GEM, was clarified to be closely associated with downregulation of the phosphorylation of IκBα. In addition, in subcutaneous tumor, liver metastatic and lung metastatic animal models, DET also amplified the inhibitory effects of GEM on tumor growth and metastasis, and these outcomes were connected with the regulation of related downstream gene products of NF-κB, such as Ki-67, PCNA and E-cadherin. In summary, DET enhanced the antitumor effect of GEM by suppressing activation of the NF-κB signaling pathway.

In addition to drug resistance, adverse reactions of GEM are another challenge during chemotherapy. Common adverse reactions to GEM include myelosuppression, peripheral neuropathy, and gastrointestinal symptoms, which often interrupt chemotherapy [50]. So far, many studies have focused on finding novel drugs with better cancer-killing effects and less toxicity, and many of these studies focus on traditional Chinese medicine. In the present study, the drug safety of DET was initially evaluated *in vivo*. Additionally, compared with GEM monotherapy, GEM combined with DET prolonged survival in mice without leading to further weight loss. These studies suggest that DET might be a novel agent for pancreatic cancer. However, there are some limitations to this study, and more studies are needed in the future. In the present study, we confirmed that DET has fewer side effects than GEM. Therefore, we speculate that it is possible to reduce the GEM dose in the combination with DET to reduce GEM side effects, increase GEM sensitivity and achieve better therapeutic effects, which is the goal of future research.

## CONCLUSIONS

In summary, our study verified that DET could inhibit cell proliferation, invasion and metastasis and induce apoptosis by interfering with mitochondrial function, inducing oxidative stress and activating caspases in pancreatic cancer. In addition, DET could promote the therapeutic effects of GEM against pancreatic cancer by inhibiting the NF-κB signaling pathway *in vitro* and *in* *vivo*. In addition, the safety of DET was preliminarily explored. Nevertheless, further research is needed to explore the function and mechanism of DET in combination therapy. In summary, our study lays the foundation for the development of DET as a novel adjuvant therapy in combination with traditional chemotherapeutic drugs to combat pancreatic cancer.

## MATERIALS AND METHODS

### Reagents and antibodies

Dulbecco’s Modified Eagle Medium (DMEM) and Roswell Park Memorial Institute (RPMI)-1640 medium were purchased from Gibco (Shanghai, China). Fetal bovine serum (FBS) was obtained from Biological Industries (Cromwell, CT, USA). Acridine orange/ ethidium bromide (AO/EB) double fluorescence staining solution was obtained from Beyotime Institute of Biotechnology (Nanjing, China). Hoechst 33342 solution and cell counting kit-8 (CCK-8) were obtained from Dojindo Molecular Technologies, Inc. (Beijing, China). Pancaspase inhibitor (z-VAD-fmk) and NF-κB inhibitor (IKK 16) were bought from Selleckchem (Shanghai, China). Anti-Bcl-2, anti-Bax, anti-cytochrome c, anti-cleaved caspase-9 and anti-cleaved caspase-3 antibodies were acquired from Cell Signaling Technology (Shanghai, China). Anti-Ki-67, anti-PCNA, anti-E-cadherin, anti-N-cadherin, anti-Snail and anti-GAPDH antibodies were obtained from Abcam (Shanghai, China). Antibodies against NF-κB-p50, NF-κB-p65, IκB-α and p-IκB-α were obtained from Santa Cruz Biotechnology, Inc. (Dallas, Texas, USA).

### Drugs

Deoxyelephantopin (Figure 1A) was acquired from BioBioPha Co., Ltd. (Kunming, China), and the purity was verified to be greater than 97%. Gemcitabine (Figure 1B) was obtained from Eli Lilly and Company (Indianapolis, Indiana, USA). Deoxyelephantopin was dissolved in DMSO to create a stock solution that was kept at -80 °C away from light. The stock solution was diluted to the working concentration with medium during the experiment. Additionally, the final concentration of DMSO in cytological experiments was less than 1‰. Gemcitabine was dissolved in sterile saline solution to create a stock solution that was kept at -80 °C.

### Cell culture

The human pancreatic cancer cell lines BxPC-3, CFPAC-1 and PANC-1 were bought from Shanghai Institute of Biological Science and Cell Resources Center, Chinese Academy of Sciences (Shanghai, China). BxPC-3 cells were cultured in RPMI-1640 medium, while CFPAC-1 and PANC-1 were both cultured in DMEM, and each medium was supplemented with 10% FBS, 100 U/mL penicillin and 100 mg/mL streptomycin. All the cells were placed in an incubator in line with typical culture conditions (37 °C, 5% CO_2_).

### Cell viability assay

The effects of DET on BxPC-3, CFPAC-1 and PANC-1 cells were identified by using the CCK-8 assay. Briefly, 5×10^3^ cells were inoculated into a 96-well microtiter plate with 100 μL of complete medium per well. The cells were cultured in a regular incubator overnight and then stimulated with DET at different concentrations for another 24 h or 48 h. Next, CCK-8 solution was added into each well (10 μl per well), and the cells were incubated for another 2 h. Finally, the absorbance was obtained by a microplate reader (Tecan, Mannedorf, Switzerland) at 450 nm. The cell viability was evaluated by the following formula: Cell viability = *A*_450_ of drug treated well/*A*_450_ of nondrug treated control well × 100

### Observation of morphological changes

BxPC-3 and CFPAC-1 cells were stimulated with DET for 24 h. Then, morphological changes were observed and photographed using light microscope (Leica, Wetzlar, Germany).

### Colony formation assay

BxPC-3 and CFPAC-1 cells were pretreated with increasing concentrations of DET and inoculated into 6-well plates at 1×10^3^ cells per well. After that, the cells were continuously cultured for approximately 12 days until clones were visible. Then, the cells were rinsed with phosphate-buffered saline solution (PBS) and fixed with cell fixing fluid (4% paraformaldehyde). Next, the cells were dyed with 1% crystal violet. The total number of visible colonies containing more than 50 cells was counted under a microscope (Olympus Corp., Tokyo, Japan).

### Fluorescent microscopy

The effect of DET on apoptosis was investigated by AO/EB and Hoechst 33342 staining following the reagent user manuals. In the AO/EB assay, 2×10^4^ cells, pretreated with drugs, were stained with AO/EB solution for 5 min. As a result of the different membrane penetration abilities of AO/EB, apoptotic cells can be distinguished from normal cells. AO reagent can enter intact cell membranes and specifically embedd in nuclear DNA, so bright green fluorescence is observed. In contrast, EB stain can only enter damaged membranes, embed nuclear DNA, and emit orange fluorescence. In the Hoechst 33342 assay, cells pretreated with DET were fixed and cocultured with Hoechst 33342 solution for 20 min. In normal cells, Hoechst 33342 can partially penetrate the membrane, showing weak blue fluorescence. In apoptotic cells, the enhanced membrane permeability and the damaged nuclear chromosome structure make reagent entry easier, resulting in strong blue fluorescence. After staining, cells were photographed under a fluorescence microscope (Leica, Washington, DC, USA)

### Apoptosis examination by flow cytometry

The effect of DET on apoptosis was further verified by an annexin V-FITC and PI double staining kit (BD Biosciences, USA). Briefly, cells were incubated in medium containing DET with or without NAC. After 24 h, cells were collected with EDTA-free trypsin, rinsed with 4 °C precooled PBS before resuspension in 500 μL precooled binding buffer. Next, the cells were dyed with 5 μL of annexin V-FITC and 5 μL of PI. Finally, the samples were filtered by 300 aperture mesh filters, and apoptosis was detected by flow cytometry (BD Biosciences, San Jose, CA, USA).

### Assessment of DET-induced oxidative stress

A reactive oxygen species assay kit (MCE, Monmouth Junction, NJ, USA) and MitoSOX red mitochondrial superoxide indicator (Thermo Scientific, Shanghai, China) were applied to assess the DET-induced oxidative stress. 2’, 7’-dichlorofluorescein-diacetate (DCFH-DA) is a special cell penetrating probe used to monitor oxidative stress. DCFH-DA itself has no fluorescence outside the cell but can cross the cell membrane and enter the cell. It can be hydrolyzed by intracellular esterase to DCFH. Intracellular nonfluorescent DCFH can be easily oxidized to DCF, which emits green fluorescence. Therefore, oxidative stress can be assessed. Cells were inoculated into black/clear flat-bottom 96-well plates (Corning, New York, USA) at 2×10^4^ cells per well in 100 μL medium and incubated overnight. Next, the previous medium was discarded, and medium containing DET with or without NAC was added. At the end of incubation, cells were rinsed with sterile PBS and coincubated with 100 μL DCFH-DA solution for 30 min at 37 °C. Eventually, DCF fluorescence intensity was detected using a fluorescence microplate reader with special reading parameters of fluorescence: 488 nm excitation wavelength and 525 nm emission wavelength.

MitoSOX, a mitochondrial fluorescent probe, can penetrate live cell membranes and specifically target mitochondria to detect superoxide in mitochondria. MitoSOX is easily oxidized by superoxide to form a specific product, emitting red fluorescence. Briefly, cells were seeded in 96-well plates overnight and stimulated with DET in the presence or absence of 100 nM MitoTEMPO (MCE, Monmouth Junction, NJ, USA). Then, the cells were coincubated with MitoSOX (5 μM) for 10 min. Finally, the cells were rinsed with prewarmed buffer, and fluorescence intensity was detected at a 510 nm excitation wavelength and a 580 nm emission wavelength.

### Measurement of mitochondrial membrane potential with JC-1

Mitochondrial membrane potential (MMP) was confirmed by a mitochondrial membrane potential kit (Merck Life Science, Shanghai, China). JC-1 is a specific probe for detecting MMP. When the MMP is at normal level, the JC-1 probe gathers in the mitochondrial matrix, forming J-aggregates and emitting light red fluorescence (525 nm excitation wavelength and 590 nm emission wavelength). Conversely, when MMP is disturbed, the JC-1 probe cannot aggregate in the mitochondrial matrix, forming a JC-1 monomer and emitting light green fluorescence (490 nm excitation wavelength and 530 nm emission wavelength). Briefly, cells were collected, resuspended in medium without serum and cocultured with JC-1 for 20 min. Finally, the intensity of the fluorescence signal of the JC-1 monomer and JC-1 polymer was tested by a fluorescence microplate reader. The MMP level was evaluated by calculating the ratio of the intensity of red and green fluorescence signals.

### Cell migration and invasion assays

Migration ability was initially measured by a wound healing assay. Briefly, a 200 μL pipette tip was used to create a straight, cell-free scratch line on a layer of cells in a petri dish. Next, the floating cells in the scratch area were washed away, and the remaining cells were cultured in serum-free conditions with or without DET. Then, the motility of each group was recorded at various time points. The migration ratio was assessed by comparing the average area of migration to the initial scratch area. Transwell assays were applied to further assess cellular motility. Briefly, cells pretreated with DET were collected, resuspended in 200 μL of medium without serum added and placed in the upper chambers of the Transwell unit with a polycarbonate filtration membrane with an 8.0 μm aperture (Corning, New York, USA). Then, 600 μL of medium containing 10% FBS was added to the lower chambers. The upper chambers with or without Matrigel (Corning, New York, USA) were used to monitor invasion and migration, respectively. After 24 h, the cells still on the upper sider of filter were rubbed off by cotton bud. Finally, cells that passed through the membranes were fixed, stained and photographed using a microscope.

### Establishing a GEM-resistant BxPC-3 cell line

In our study, the GEM-resistant cell line was established by continuously stimulating with cumulative concentrations of GEM ranging from 10 nM to 100 nM for 25 weeks as previously described [51, 52]. Briefly, BxPC-3 cells were seeded into a 25 cm^2^ culture flask. When cells were healthy and the confluence reached 80%, medium containing 10 nM GEM was added, and cells were continuously cultured until significant cell death occurred. Next, the medium was replaced with drug-free medium. The surviving cells were further cultured and passaged, and the GEM concentration gradually increased according to the cell growth conditions. The above operations were repeated. Finally, cells resistant to GEM of 50 nM and 100 nM (BxPC-3/GEM50 and BxPC-3/GEM100) were selected, expanded and frozen for subsequent experiments. To ensure good reliability of GEM-resistant cell lines, a new cryopreserved vial of cells was resuscitated every 2-3 months, and monthly mycoplasma tests were conducted to ensure mycoplasma-negative cultures.

### Measurement of intracellular glutathione

Intracellular glutathione (GSH), an important endogenous antioxidant and free radical scavenger, is associated with oxidation resistance. In the case of oxidative stress, reduced GSH is depleted and converted to the oxidized form (GSSG); therefore, the ratio of GSH to GSSG is an indicator of oxidative stress. In our experiment, the levels of reduced GSH and GSSG were analyzed by using a GSSG/GSH quantification kit (Dojindo, Beijing, China). Briefly, cells were stimulated with DET for 24 h. Then, the total GSH and GSSG levels were detected at a wavelength of 412 nm. The level of reduced GSH was calculated according to the following formula: GSH = Total Glutathione (GSH + GSSG) – 2 × GSSG. Cellular redox state was evaluated through the ratio of GSH to GSSG.

### Measurement of the activation of intracellular thioredoxin reductase

Thioredoxin reductase (TrxR), an NADPH-dependent dimer selenium-enzyme containing FAD domain, is the pivotal enzyme of the Trx antioxidant system. In this study, intracellular TrxR activity was monitored using a thioredoxin reductase assay kit (Solarbio, Beijing, China). Briefly, cells treated with DET for 24 h were lysed by RIPA lysis buffer. Then, the mixture was centrifuged at 15,000 g for 15 min at 4 °C, and the supernatant was collected in Eppendorf tubes. The total soluble protein concentration was measured by using a BCA protein assay kit (Thermo Scientific, Shanghai, China). Finally, 100 μL of protein sample (together with 900 μL of reagent) was used to detect intracellular TrxR activity at a wavelength of 412 nm. A unit of TrxR activity is defined as 1 mg of thioredoxin reductase that catalyzes the reduction of 1 nmol DTNB to an enzyme activity unit at 25 °C per min; this is calculated with the following formula:

$$\begin{array}{c} \text{TrxR}\;(\text{U/mg}\;\text{prot})=(\Delta A_{\text{sample}}-\Delta A_{\text{blank}})/(\varepsilon \times \text{d})\times 10^{9}\\ \;\;\times {\text{V}}_{\text{total}}/(\text{Cpr}\times {\text{V}}_{\text{sample}})/\text{T}\\ =147\times (\Delta A_{\text{sample}}-\Delta A_{\text{blank}})/\text{Cpr} \end{array}$$

ε: the molar extinction coefficient of TNB at 412 nm, 1.36 × 10^4^ L/mol/cm; d: the length of the optical path of cuvette, 1.0 cm; V_total_: total volume of reaction system, 1 × 10^-3^ L; Cpr: protein concentration of supernatant (mg/mL), obtained by BCA assay; V_sample_: supernatant volume added into the reaction system, 0.1 mL; T: reaction time, 5 min; Δ*A*: absorbance at 412 nm.

### Immunoblotting

Cells pretreated with DET for 24 h were rinsed with ice-cold PBS and lysed in lysis buffer containing 1 mM phenylmethylsulfonyl fluoride and phosphatase inhibitor on ice. Then, the lysed cells were scraped with a cell scraper, and the mixture was collected into 1.5 mL Eppendorf tubes and centrifuged at 15,000 g 4 °C for 15 min. The supernatant was recollected in new Eppendorf tubes. Nuclear proteins and cytoplasmic proteins were separated by using a nuclear and cytosol fractionation kit (Thermo Scientific, Shanghai, China), and the protein concentration was analyzed by using a BCA kit. The extraction procedure for tumor tissue protein was similar to that for cells. The proteins (total mass: 30 or 50 micrograms) were electrophoresed on SDS-PAGE gels ranging from 8% to 12%. Next, proteins were transferred to 0.45 μm PVDF membranes preactivated with methanol (Millipore, USA). Then, the PVDF membranes were blocked with skimmed milk (2.5 g milk powder dissolved in 50 mL TBST) in a constant temperature shaker at 37 °C for 2 h. After that, the PVDF membranes were coincubated with different primary antibodies overnight at 4 °C, including those targeting Bax (dilution ratio: 1/1,000), Bcl-2 (dilution ratio: 1/1,000), cleaved caspase-9 (dilution ratio: 1/1,000), cleaved caspase-3 (dilution ratio: 1/1,000), cytochrome c (dilution ratio: 1/1,000), IκB-α (dilution ratio: 1/500), p-IκB-α (dilution ratio: 1/500), NF-κB-p50 (dilution ratio: 1/800), NF-κB-p65 (dilution ratio: 1/800), E-cadherin (dilution ratio: 1/500), N-cadherin (dilution ratio: 1/1,000), Snail (dilution ratio: 1/1,000) and GAPDH (dilution ratio: 1/10,000). After that, the membranes were washed with TBST and incubated with secondary antibody (CST, Danvers, MA, USA) at 22-25 °C for 1 h. Finally, the target protein bands were observed and photographed with an enhanced chemiluminescence kit (ECL, Keygen, Nanjing, China).

### *In vivo* growth and metastasis study

Our animal experiments conformed to the institutional guidelines of the Animal Health Care and Use Committee of Harbin Medical University. Nude mice (BALB/cAnNCrj-nu, aged 4-6 weeks) purchased from Charles River (Beijing, China) were used. Mice were kept in a specific pathogen-free (SPF) system with water and food certified by SPF level. To confirm the inhibition of tumor growth by DET and GEM, 100 μL of BxPC-3 cell suspension (5×10^7^ cells/mL) was administered subcutaneously into the right middle armpit of mice to establish subcutaneous tumors. Tumor size was recorded every 3 days using a caliper, and the tumor volume was calculated by using the following formula: V = (π/6) × L × W^2^ (V, volume; L, length diameter; W, width diameter). When the volume approached approximately 100 mm^3^, mice were randomly assigned to four groups (4 per group): control group (DMSO dissolved in PBS), DET group (10 mg/kg, i.p. every two days), GEM group (100 mg/kg, i.p. twice a week), and GEM combined the DET group (according to the above dosage). The doses of DET and GEM used in the present study were in accordance with previous studies [53]. The mice were euthanized after carefully fed and monitored for 33 days. The tumor specimens were removed and divided into two parts: one was stored at -80 °C, and the other was fixed in paraformaldehyde for further research. A liver metastatic model and a lung metastatic model were designed to verify the effect of DET on metastasis. In the liver metastasis model, a 1.5 cm surgical incision was made in the upper left lateral abdomen of mice, and the spleen was found and exposed. After that, 1×10^6^ BxPC-3 cells were injected into the distal part of the spleen and a gel sponge and cotton swab were used for hemostasis. Then, the mice were divided into four groups and treated with the same strategies as above. After 54 days of feeding and observation, the mice were sacrificed, and the livers were dissected to evaluate the tumor metastasis. In a lung metastasis model, 200 μL of BxPC-3 cells (luciferase-labeled) suspension (2×10^7^ cells/mL) were inoculated into mice via the tail vein. Next, the mice were assigned to treatment groups as described above. After continued treatment and monitoring for 6 weeks, the mice were imaged using the *In-Vivo* Xtreme system (Bruker Scientific Technology, Germany). After that, mice were euthanized by cervical dislocation, and the lungs were dissected to identify the extent of metastasis. Other organs, including hearts, kidneys and livers, were also dissected and fixed in paraformaldehyde for further histological analysis. Furthermore, the effects of drugs on body weight and survival time were initially evaluated using a lung metastasis model. The body weight was recorded every 7 days, and the survival was observed every 4 days. These operations were maintained constantly until the end of the experiment.

### Histology and immunohistochemistry

Subcutaneous tumors, livers, lungs and other organs fixed in paraformaldehyde were embedded in paraffin. Then, the paraffin-embedded organs were sectioned (5 μm) for (H&E) staining to evaluate the inhibition of DET on pulmonary metastasis and potential side effects. Paraffin-embedded subcutaneous tumors were submitted to immunohistochemical staining with Ki-67, PCNA and E-cadherin antibodies. The total number of positively stained cells (nuclear stained in brown) was calculated by randomly selecting 10 microscopic fields (×200).

### Data analysis

GraphPad Prism 8.02 software and SPSS 25.0 software were applied to complete the statistical analysis. Quantitative data of the test are represented as the mean ± S.D. Statistically significant differences among treatment groups were confirmed by ANOVA and *t*-test. *P*<0.05 was defined as statistically significant and indicated by different statistical notations. A log-rank test was used to evaluate the Kaplan–Meier survival curves of mice.

## Supplementary Material

Supplementary Figure 1
