# Supplementary material for: Deoxyelephantopin induces apoptosis via oxidative stress and enhances gemcitabine sensitivity in vitro and in vivo through targeting the NF-κB signaling pathway in pancreatic cancer
Source: Aging (Albany NY). 2020 Jun 11;12(11):11116–38. doi: 10.18632/aging.103327 (PMC7346037; doi:10.18632/aging.103327)
Supplement: Supplementary Figure 1 [file aging-12-103327-s001..pdf]

SUPPLEMENTARY FIGURE

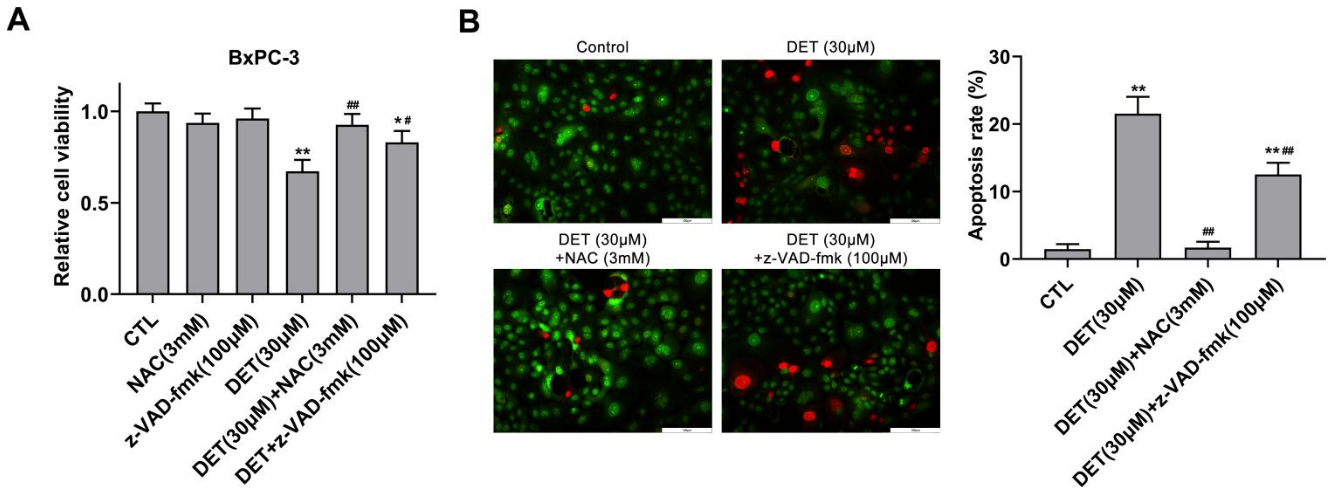

Supplementary Figure 1. NAC and z-VAD-fmk inhibited DET-induced apoptosis in BxPC-3 cell. (**A**) Pretreatment with NAC and z-VAD-fmk rescued DET-induced decline in cell viability, as shown by CCK-8 assay. **P* < 0.05, ***P* < 0.01 versus CTL. #*P* < 0.05, ##*P* < 0.01 versus DET (30 μM) single treatment group. CTL, control. (**B**) NAC and z-VAD-fmk relieved DET-induced apoptosis in BxPC-3 cells, shown by AO/EB double staining assay. ***P* < 0.01 versus CTL. ##*P* < 0.01 versus DET (30 μM) single treatment group. CTL, control. Magnification, × 200 (**B**). Scale bar, 100 μm (**B**). Data are shown as mean ± SD of at least three independent experiments.
